# Design and Fabrication of Capacitive Silicon Nanomechanical Resonators with Selective Vibration of a High-Order Mode

**DOI:** 10.3390/mi8100312

**Published:** 2017-10-20

**Authors:** Nguyen Van Toan, Tsuyoshi Shimazaki, Naoki Inomata, Yunheub Song, Takahito Ono

**Affiliations:** 1 Graduate School of Engineering, Tohoku University, Sendai 980-8579, Japan; shimazaki@nme.mech.tohoku.ac.jp (T.S.); inomata@nme.mech.tohoku.ac.jp (N.I.); ono@nme.mech.tohoku.ac.jp (T.O.); 2 Department of Electronic Engineering, Hanyang University, Seoul 04763, Korea; yhsong2008@hanyang.ac.kr

**Keywords:** capacitive silicon resonator, nanomechanical resonator, selective vibration, and high-order mode

## Abstract

This paper reports the design and fabrication of capacitive silicon nanomechanical resonators with the selective vibration of a high-order mode. Fixed-fixed beam capacitive silicon resonators have been successfully produced by the use of electron beam lithography, photolithography, deep reactive ion etching, and anodic bonding methods. All resonators with different vibration modes are designed to have the same resonant frequency for performance comparison. Measurement results show that higher-order mode capacitive silicon resonators can achieve lower insertion loss compared to that of lower-order mode capacitive silicon resonators. The motional resistance of the fourth mode vibration resonator is improved by 83%, 90%, and 93% over the third, second, and first mode vibration resonators, respectively.

## 1. Introduction

Many IoT (internet of things) devices can be connected to the internet via a wireless network [1]. Increasing the amount of transmitted and received information and requiring the accurate data transmissions are necessaries. Satisfying these issues, micro clock generators for transmitters and receivers with a smaller size and higher performance are required.

Quartz crystal resonators are usually employed for the above applications, as they exhibit high-quality factor [2,3], good power handling [4], and excellent temperature stability [5,6]. However, their vibration is on a small scale owing to the direct physical contact between the electrodes and the resonant body. Their working frequencies, dependent on the thickness of a piezoelectric film, are in a low range frequency. Also, their fabrication process is not compatible with complementary metal-oxide-semiconductor (CMOS) fabrication. Capacitive silicon resonators, on the other hand, are expected to overcome the above problems, as has been presented in many works [7,8,9,10,11,12,13]. An ultra-high *Q* factor can be achieved by capacitive silicon resonators, as reported in References [7,8,9]. In addition, they are capable of integration with a CMOS chip [10] and they exhibit excellent long-term stability [7,8]. Their resonant frequency (fundamental mode) depends on the geometric dimensions of the resonators [7,13]. For instance, resonant frequencies of bar-type [13], square-type [12], and disk-type [9] capacitive silicon resonators are decided by the width of the resonant body. A fixed-fixed beam capacitive silicon resonator, such as that presented in Reference [7], contains the resonant beam body, as well as driving and sensing electrodes. Its resonant frequency is designed by its length and width. In attaining high-frequency capacitive silicon resonators, downscaling (reducing the length and width of the resonant body) is a common solution, although this induces problems such a large motional resistance and high insertion loss. The motional resistance of resonators is always desired to be as low as possible for an impedance that matches the CMOS chip. Hence, the downscaling method makes capacitive silicon resonators hard to apply to practical applications (such as integration with electrical circuits). Pursuant to an increase of the operating frequency without downscaling the resonant structures, this work focuses on capacitive silicon nanomechanical resonators that are able to vibrate at a higher mode selectively based on placing the driving electrodes along the resonant body. The first, second, third, and fourth mode fixed-fixed beam capacitive silicon resonators are produced and examined.

## 2. Device Description

**Figure 1:**
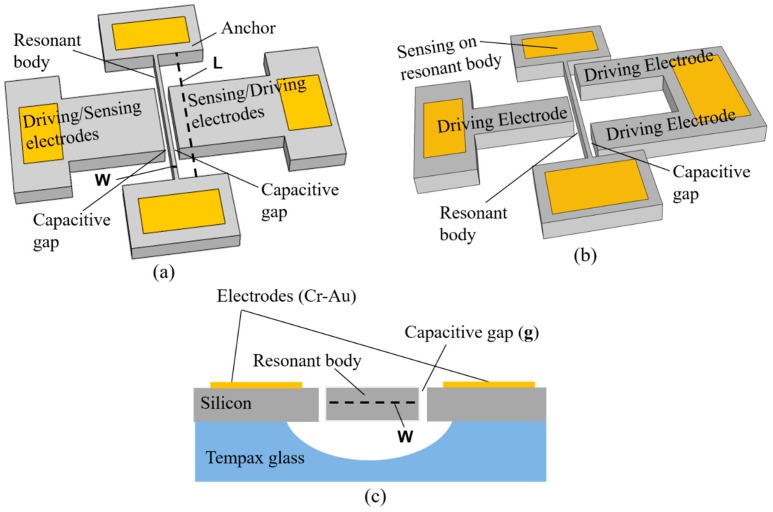
Fixed-fixed beam capacitive silicon resonators. (**a**) First mode vibration structure; (**b**) third mode vibration structure; (**c**) cross-sectional structure.

Figure 1a,b present a perspective-view schematic of the fixed-fixed beam capacitive silicon resonators with first and third mode vibration, respectively. The basic components of resonator structures are the resonant body, capacitive gaps, the driving electrode, and the sensing electrode. The resonant body is suspended by the two anchors at each end of the resonant body on the patterned glass substrate. The cross-sectional view of the resonator structures is shown in Figure 1c. For the first mode vibration structure, the driving electrode is placed along the side of the resonant body and the sensing electrode is placed in another side of the resonant body, as shown in Figure 1a. They are separated from the resonant body by narrow capacitive gaps. In turn, for the third mode vibration, the driving electrodes are designed and placed on the both sides of the resonant body and the sensing electrode, as the motional detection is on the resonant body electrode (Figure 1b). The high-order mode vibration structures are decided by the number of the driving electrodes along the resonant body. The number of driving electrodes for the second, third, and fourth mode vibration is 2, 3, and 4, respectively.

To operate the resonators, an AC input signal *V*_AC_ together with DC bias voltage *V*_DC_ are applied to a driving electrode, which results in an electrostatic force that acts on the resonant body vibration. This motion results in the changes of the motional capacitance of the resonators owing to the changes in the size of the capacitive gaps. Based on monitoring in a time-varying electrostatic force, the resonant frequency of the resonators can be observed.

The resonant body is actuated by a delta deviance electrostatic force, which is generated by the combined effects of DC voltage and AC voltage, given as:

(1) $$\Delta F=\frac{\varepsilon_{r}\varepsilon_{0}A_{el}}{g^{2}}V_{\mathrm{DC}}V_{\mathrm{AC}}$$

where *A_el_* is the area of the electrode plate, *ε_r_* is the dielectric constant of the material between the plates (for an air environment, *ε_r_* ≈ 1), *ε*_0_ is the electric constant (*ε*_0_ ≈ 8.854 × 10^−12^ F·m^−1^), and *g* is the distance between two plates called the capacitive gap.

The resonant frequencies *f_n_* are determined by the formula of the effective spring constant *k_eff_* and the effective mass *m_eff_*, as follows:

(2) $$f_{n}=\frac{1}{2\pi }\sqrt{\frac{k_{eff}}{m_{eff}}}$$

The effective spring constant and effective mass of the resonators are given by:

(3) $$k_{eff}=\lambda_{n}^{4}n\frac{EI_{z}}{L^{3}}$$

(4) $$m_{eff}=nm_{0}$$

where *λ_n_* is the frequency coefficient for each resonance mode, *E* is the Young’s modulus of the resonator material, *I_z_* is the area moment of inertia, *L* is the length of the resonant body, and *m*_0_ is the mass of the resonators.

Equations (2)–(4) can be combined into the equation below [14]:

(5) $$f_{n}=k_{n}\frac{W}{L}\sqrt{\frac{E}{\rho }}$$

where *k_n_* is the corresponding constant value for each resonance mode and *ρ* is the density of the structure material. The *k_n_* values for the first, second, third, and fourth resonance modes are *k*_1_ = 1.027, *k*_2_ = 2.833, *k*_3_ = 5.54, and *k*_4_ = 9.182, respectively.

The equivalent circuit model of the capacitive resonators is reported in many works [10,11,12], and consisted of the motional resistance *R*_m_, motional inductance *L*_m_, motional capacitance *C*_m_, and feed-through capacitance *C_f_*.

(6) $$L_{m}=\frac{m_{eff}}{\eta^{2}}$$

(7) $$C_{m}=\frac{\eta^{2}}{k_{eff}}$$

(8) $$R_{m}=\frac{\sqrt{k_{eff}m_{eff}}}{Q\eta^{2}}$$

(9) $$\eta =V_{\mathrm{DC}}\varepsilon_{0}\frac{Lt}{g}$$

where *Q* is the quality factor of the resonator, *t* is the thickness of the resonator, and *η* is an electromechanical transduction factor.

The larger the transduction factor *η* in the resonator, the more electrical energy will be converted into the mechanical domain, and consequently the bigger the force difference that can be gained for the vibration in the capacitive resonators. In this work, the high-order mode vibration has a longer resonant body compared to that of other resonators. This means that its transduction factor is larger, which results in a greater chance of capacitance compared to others. Thus, a high vibration peak (low insertion loss) can be achieved. Also, the small motional resistance can be expected (the motional resistance is proportional to the second order of the transduction factor (Equation (8)).

The motional resistance of resonators can be calculated by Equation (3); however, this equation becomes complex when considered at the high-order mode vibration. Another way to estimate the motional resistance of resonators is based on the insertion loss, which is not dependent on the vibration mode of structures, as follows [15]:

(10) $$R_{m}=50\left(10^{\frac{IL_{dB}}{20}}-1\right)$$

where *IL_dB_* is the insertion loss of the transmission and its unit is in decibels (dB).

**Figure 2:**
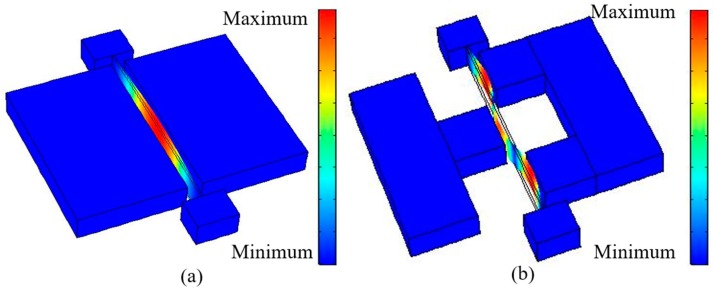
Finite element method (FEM) simulation. (**a**) First mode vibration; (**b**) third mode vibration.

The fixed-fixed beam resonators presented in this work are designed for lateral vibration as a flexural mode. A finite element method model is built by COMSOL (Version 5.2a, Keisoku Engineering System Co., Ltd, Tokyo, Japan) for a prediction of the vibration shape and the resonant frequency. Figure 2a,b show the vibration shapes of the first and third modes, respectively. The colors correspond to total in-plane displacement, where red denotes the maximum displacement and blue represents no displacement. Other vibration mode shapes can be found in Table 1.

In this work, all resonators are designed to have the same resonant frequency for performance comparison. The fixed-fixed beam resonators with the first, second, third, and fourth mode vibrations are designed and fabricated. The resonator parameters, their theoretical calculations, and finite element method (FEM) simulations are shown in Table 1.

## 3. Experiments

In this section, the fabrication process, measurement setups, and measurement results are presented. Devices are produced by electron beam (EB) lithography, photolithography, deep reactive ion etching (RIE), and anodic bonding methods. To save time during the exposing process of EB lithography, only nano capacitive gaps are formed. An extra conventional photolithography is subsequently performed to create the resonator structures. The formed silicon resonator structures on a silicon on insulator (SOI) wafer are transferred to a glass substrate by anodic bonding in order to reduce the parasitic capacitances of the handling silicon layer. Detailed information on the procedure follows.

### 3.1. Experimental Methodology

**Figure 3:**
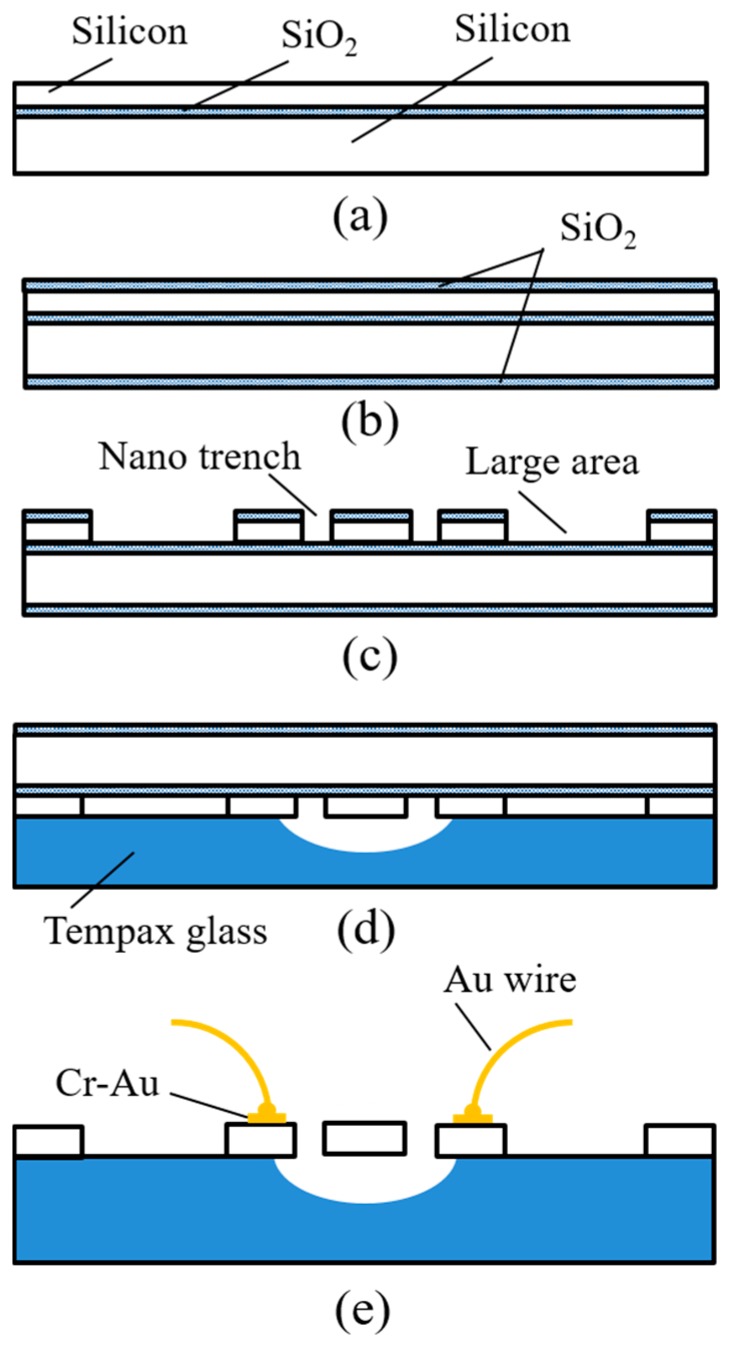
Fabrication process. (**a**) Silicon on insulator (SOI) wafer (7 μm/ 1 μm/ 300 μm); (**b**) thermal oxidation; (**c**) combination of electron beam (EB) lithography, photolithography, and deep reactive ion etching (RIE) process; (**d**) anodic bonding; (**e**) backside silicon etching, SiO_2_ removal, and metal contact pads.

**Figure 4:**
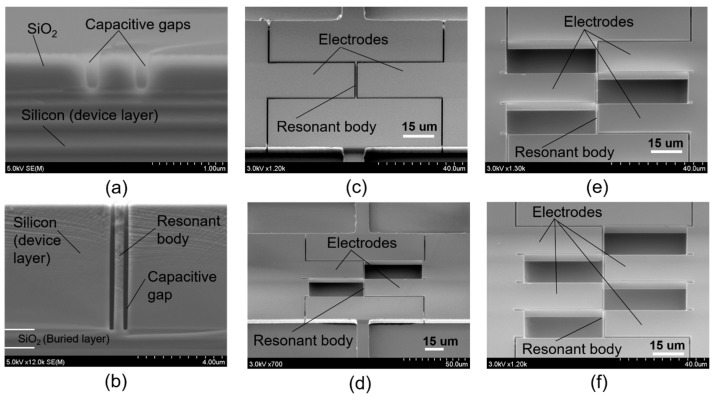
Fabricated results. (**a**) SiO_2_ patterning with EB resist and using the RIE technique; (**b**) resonant body and narrow trenches formed by deep RIE; (**c**) first mode vibration structure; (**d**) second mode vibration structure; (**e**) third mode vibration structure; (**f**) fourth mode vibration structure.

The starting substrate is an SOI wafer with a 7-μm-thick device layer with a low resistivity of 0.02 Ωcm, a 1-μm-thick oxide layer, and a 300-μm-thick silicon handling layer (Figure 3a). After conventional cleaning including RCA1, RCA2, and piranha, an approximately 500-nm-thick SiO_2_ layer is formed on the entire surface of the SOI wafer via wet thermal oxidation (Figure 3b). Then, a 400-nm-thick EB resist (ZEP 502A) is patterned on the above SiO_2_ layer (on the device layer side). The reactive ion etching (RIE) method is employed to etch SiO_2_ with the EB resist as a mask, using a gas mixture of CHF_3_ and Ar with a power of 120 W and a chamber pressure of 5 Pa. Narrow gaps with smooth and vertical etched shapes were achieved, as shown in Figure 4a. After removing the EB resist, the nano trenches on the top silicon layer are then formed by the deep RIE with Bosch process using SF_6_ (etching cycles of 2.5 s) and C_4_F_8_ (passivation cycles of 2.5 s) gases. Figure 4b shows the nano trenches formed using the above process. The resonant body and capacitive gaps are 500 nm and 300 nm, respectively. Following this, the resonator structures are created by employing photolithography following the deep RIE of silicon (Figure 3c).

To reduce the parasitic capacitances from the handling silicon substrate, the 300-μm-thick Tempax glass (Shibuya Optical Co., Ltd, Wako-Shi, Saitama, Japan) substrate is employed for the transferring process. The resonator structures on the SOI wafer are aligned and bonded with the Tempax glass substrate by an anodic bonding technique (Figure 3d). The bonding process is performed at 400 °C with 800 V power sources for 15 min. Although the thermal expansion coefficients of Si and glass are similar, they possibly cause stress on silicon device layer after bonding. To avoid this problem, the cooling process is important. It takes few hours to complete the cooling process. The heated stage is decreased slowly. The backside silicon handling layer is removed by the plasma etching of SF_6_ gas. After the buried SiO_2_ layer is etched out by buffered hydrofluoric acid (BHF) solution and devices are dried by a supercritical CO_2_ process to avoid sticking issues, the electrode pads using Cr-Au are formed by a sputtering process via a shadow mask. Finally, the Au wire bonding process is conducted, as shown in Figure 3e.

Figure 4c–f show the successfully fabricated devices with differently expected mode vibration shapes, including the first, second, third, and fourth mode, respectively.

### 3.2. Measurement Setup

**Figure 5:**
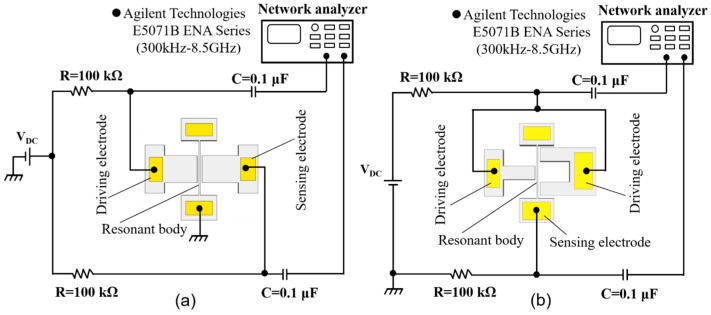
Measurement setups. (**a**) First mode vibration structure: (**b**) high-order mode vibration structure.

The frequency responses of the fabricated devices are evaluated by the electrical setup shown in Figure 5. The measurement setup contains a network analyzer (E5071B ENA Series, Agilent Technologies, Santa Clara, CA, USA) with a frequency range from 300 kHz to 8.5 GHz, DC voltage source *V*_DC_, electrical components including capacitors and resistors, and coaxial cables. The resonators are set in a vacuum chamber at a pressure chamber of 0.01 Pa.

To detect the motion of the first mode vibration structure, the setup shown in Figure 5a is employed. The output and input ports of the network analyzer are connected to the driving and sensing electrodes, respectively, through the resistors and capacitors, while the resonant body electrode is attached to the ground. The purpose of usage of the resistors and capacitors is to decouple the radio frequency (RF) signal and also to avoid damaging the network analyzer. In turn, to the high-order mode vibration, all driving electrodes on both sides of the resonant body electrode are connected to the output port of the network analyzer while the resonant body is connected to the input port of the network analyzer (Figure 5b).

### 3.3. Measurement Results

**Figure 6:**
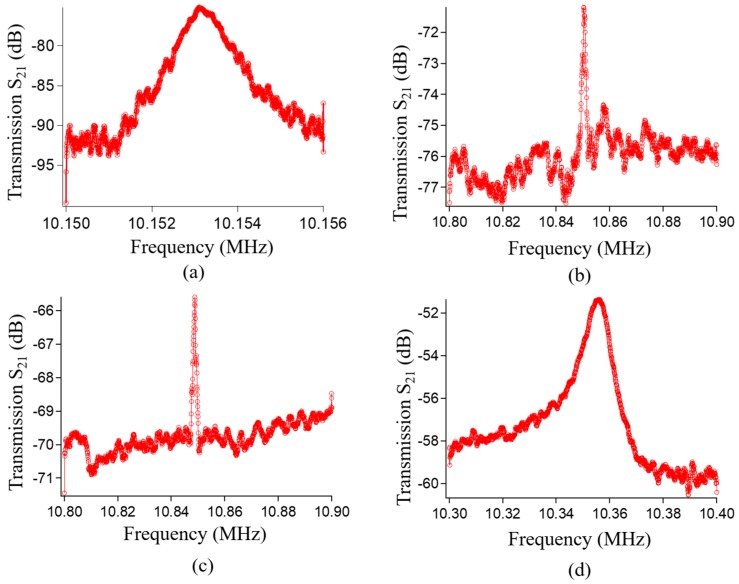
Frequency responses. (**a**) First mode vibration structure; (**b**) second mode vibration structure; (**c**) third mode vibration structure; (**d**) fourth mode vibration structure.

The specifications of the fabricated devices are summarized in Table 2. The frequency responses of the fabricated devices are shown in Figure 6. The evaluation conditions are the same for all fabricated devices under *V*_DC_ = 15 V, *V*_AC_ = 0 dBm and a vacuum chamber of 0.01 Pa. Similar resonant frequency values are observed for all fabricated devices, although their resonant lengths are significantly different (Table 1). Thus, by placing the driving electrodes along the resonant body, the high-order mode capacitive resonators can be demonstrated.

The resonant peak of the first mode vibration structure is found at 10.15 MHz with a quality factor *Q* of 10,000, as shown in Figure 6a. The measured resonant frequency is in good agreement with the FEM simulation result (Table 1). Figure 6b–d show the frequency responses of the second, third, and fourth mode vibration structures, respectively. No other vibration modes were observed in these resonators.

The quality factor decreases as the vibration mode increases. A possible reason for this is the large supporting loss [16,17] and thermoplastic damping [18,19,20] of the high-order mode compared to those of the lower modes, which result in a high energy dissipation. Nevertheless, the insertion loss of the fabricated devices is improved from −75 dB to −51.5 dB as the vibration modes are raised from the first to fourth mode capacitive devices. The motional resistance of the first mode vibration structure is 281 kΩ, while that of the second, third, and fourth mode vibration structures is 181, 95, and 18.7 kΩ, respectively. Thus, the motional resistance of THE fourth mode vibration structure is reduced by 83%, 90%, and 93% over the third, second, and first mode vibration structures, respectively.

The methods to reduce the motional resistance while maintaining the high *Q* factor comparable to that of the first mode vibration structure are suggested as follows: low supporting loss by U-shaped supports instead of straight supports [21], low damage etched surfaces by the choice of fabrication technologies [22], and low thermoplastic dissipation by optimizing the design [12].

## 4. Conclusions

In this work, high-order mode capacitive silicon resonators are produced by placing the driving electrodes along the resonant body, and the fabricated resonators are examined. It is demonstrated that the higher-order mode resonators can achieve lower insertion loss and smaller motional resistance compared to lower-order mode resonators. Not only fixed-fixed beam capacitive silicon resonators, but also other types of capacitive silicon resonators including bar-type, disk-type, and square-type could be employed in this proposal for the production of high-order vibration modes with low motional resistance.

## Figures and Tables

**Table 1:** Summary of parameters of the first, second, third, and fourth mode capacitive resonators.

Resonator Structures	Vibration Modes	First Mode	Second Mode	Third Mode	Fourth Mode
Parameters	Resonant length	21.3 μm	35.5 μm	49.3 μm	63.3 μm
	Resonant width	0.5 μm	0.5 μm	0.5 μm	0.5 μm
	Resonant thickness	7 μm	7 μm	7 μm	7 μm
	Capacitive gap	0.3 μm	0.3 μm	0.3 μm	0.3 μm
	Number of driving electrodes	1	2	3	4
Calculation	Frequency	9.66 MHz	9.60 MHz	9.73 MHz	9.79 MHz
Finite element method (FEM) Simulation	Frequency	9.71 MHz	9.68 MHz	9.73 MHz	9.78 MHz
	Vibration mode (resonant body only)	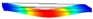	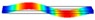	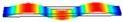	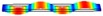

**Table 2:** Summary of measurement conditions and evaluation results of the first, second, third, and fourth mode capacitive resonators.

Resonator Structures	Vibration Modes	First Mode	Second Mode	Third Mode	Fourth Mode
Measurement conditions	*V*_AC_	0 dBm	0 dBm	0 dBm	0 dBm
	*V*_DC_	15 V	15 V	15 V	15 V
	Pressure level	0.01 Pa	0.01 Pa	0.01 Pa	0.01 Pa
Experimental results	Resonant frequency	10.15 MHz	10.85 MHz	10.85 MHz	10.36 MHz
	Quality factor	10078	8768	4255	844
	Insertion loss	−75 dB	−71 dB	−65.6 dB	−51.5 dB
	Motional resistance	281 kΩ	181 kΩ	95 kΩ	18.7 kΩ
